# Lung ILC2s are activated in BALB/c mice born to immunized mothers despite complete protection against respiratory syncytial virus

**DOI:** 10.3389/fimmu.2024.1374818

**Published:** 2024-05-17

**Authors:** Jessica L. Kosanovich, Katherine M. Eichinger, Madeline A. Lipp, Sonal V. Gidwani, Devarshi Brahmbhatt, Mark A. Yondola, David H. Chi, Timothy N. Perkins, Kerry M. Empey

**Affiliations:** ^1^ Department of Pharmaceutical Sciences, University of Pittsburgh School of Pharmacy, University of Pittsburgh, Pittsburgh, PA, United States; ^2^ Center for Clinical Pharmaceutical Sciences, University of Pittsburgh School of Pharmacy, University of Pittsburgh, Pittsburgh, PA, United States; ^3^ Calder Biosciences Inc., New York, NY, United States; ^4^ Division of Pediatric Otolaryngology, Children’s Hospital of Pittsburgh, University of Pittsburgh School of Medicine, Pittsburgh PA, United States; ^5^ Department of Pathology, University of Pittsburgh School of Medicine, Pittsburgh, PA, United States; ^6^ Department of Pharmacy and Therapeutics, University of Pittsburgh School of Pharmacy, University of Pittsburgh, Pittsburgh, PA, United States; ^7^ Department of Immunology, University of Pittsburgh School of Medicine, University of Pittsburgh, Pittsburgh, PA, United States

**Keywords:** RSV, maternal immunization, ILC2, Fcgamma receptors, IL-33

## Abstract

Activated lung ILC2s produce large quantities of IL-5 and IL-13 that contribute to eosinophilic inflammation and mucus production following respiratory syncytial virus infection (RSV). The current understanding of ILC2 activation during RSV infection, is that ILC2s are activated by alarmins, including IL-33, released from airway epithelial cells in response to viral-mediated damage. Thus, high levels of RSV neutralizing maternal antibody generated from maternal immunization would be expected to reduce IL-33 production and mitigate ILC2 activation. Here we report that lung ILC2s from mice born to RSV-immunized dams become activated despite undetectable RSV replication. We also report, for the first time, expression of activating and inhibitory Fcgamma receptors on ILC2s that are differentially expressed in offspring born to immunized versus unimmunized dams. Alternatively, ex vivo IL-33-mediated activation of ILC2s was mitigated following the addition of antibody: antigen immune complexes. Further studies are needed to confirm the role of Fcgamma receptor ligation by immune complexes as an alternative mechanism of ILC2 regulation in RSV-associated eosinophilic lung inflammation.

## 1 Introduction

The neonatal period of lung development is a formative timeframe responsible for establishing a tissue resident ILC2 pool that persists through adulthood (1, 2). ILC2 precursors seed the lungs during prenatal development and expand to adult levels in the first 2 weeks of life. During this time, ILC2s adopt a lung-specific transcriptional signature that dictates subsequent activation, expansion, and maintenance (1). These neonatally-established ILC2s constitute the majority of the adult ILC2 pool and represent the predominant ILC2 population that expands in response to stimulation (1). Importantly, ILC2s trained during neonatal activation are more functionally competent upon re-stimulation, demonstrating more vigorous responses to stimulation than newly established adult ILC2s (2). Consistent with this work, we previously identified a hyperresponsive ILC2 (hILC2) population in maternally vaccinated offspring following secondary exposure to respiratory syncytial virus (RSV) (3). As a prior stimulation is necessary to “train” a more functionally competent ILC2 population in adulthood, the presence of hILC2s in offspring born to dams immunized with an adjuvated prefusion RSV F vaccine, led us to theorize that ILC2s were activated during primary neonatal RSV exposure in the presence of RSV-neutralizing maternal antibody (matAb). Here we describe the role of antibody:antigen immune complexes in regulating lung ILC2 activation and propose this as an alternative mechanism by which ILC2s may be activated in offspring born to RSV-immunized dams.

## 2 Materials and methods

### 2.1 Maternal immunization, intranasal RSV infections, and treatments

**Figure 1:**
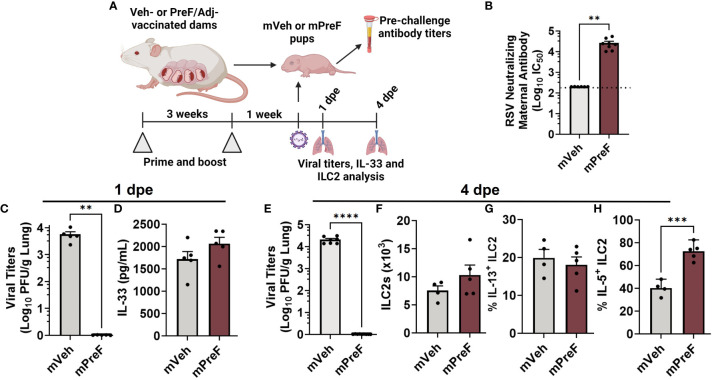
ILC2 activation occurs in mPreF pups despite complete protection from replicating RSV and similar levels of IL-33. 1 week prior to parturition, pregnant Balb/c mice completed a 2-dose vaccination series of vehicle (mVeh) or PreF/Adjuvant (mPreF). At PND6, a cohort of mVeh and mPreF pups were culled for pre-challenge serum antibody analysis while a second cohort of pups were intranasally exposed to 5x10^5^ PFU/gm RSV L19 and culled at 4dpe for viral titer and ILC2 analysis **(A)**. Pre-challenge serum from mVeh and mPreF pups was analyzed for RSV neutralizing antibody **(B)**. At 1dpe, left lungs were analyzed for viral titers **(C)** and right lung homogenate was processed for IL-33 quantification **(D)**. At 4dpe, left lungs were analyzed for viral titers **(E)**, while total lung ILC2s **(F)** and frequency of IL-13^+^ ILC2s **(G)** and IL-5^+^ ILC2s **(H)** were analyzed from the right lungs. Pups were born to dams immunized with preF/Advax **(B–D)** or preF/Alum (**E–H**). Data are represented as mean ± SEM (n=4-8 mice per group). Nonlinear regression was used to obtain IC50 values with the dotted line indicating the lower limit of quantification **(B)**. Statistical significance was calculated using an unpaired t-test. **p ≤ 0.01, ***p ≤ 0.001, and ****p ≤ 0.0001 (**C-H**). Created with BioRender.com.

**Figure 2:**
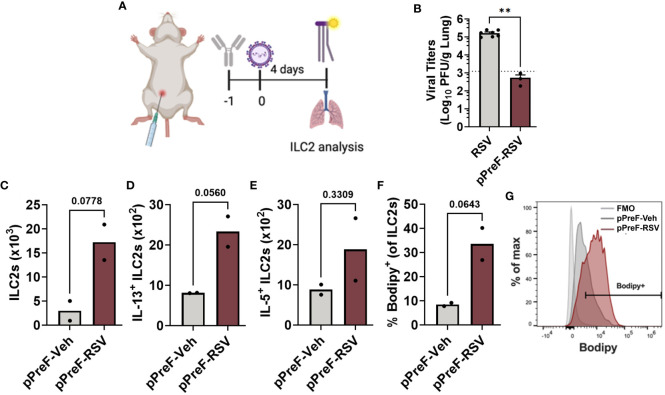
Passive transfer of RSV-neutralizing antibody leads to ILC2 activation. Adult Balb/cJ mice were administered heat-inactivated serum from PreF/Alum-vaccinated dams one day prior to intranasal exposure to MEM (pPreF-Veh) or 5x10^5^ PFU/gm RSV L19 (pPreF-RSV). At 4dpe, mice received an intraperitoneal injection of Bodipy FL C16 one hour prior to sacrifice, at which time lungs were harvested for ILC2 analysis **(A)**. Left lungs were collected from non-immunized (RSV) and passively immunized (pPreF-RSV) adult mice for viral titer analysis **(B)**. Dotted line represents the lower limit of viral quantification defined as less than five plaques in a well. Total ILC2s **(C)**, IL-13+ ILC2s **(D)** and IL-5+ ILC2s **(E)**, as well as the frequency of Bodipy+ ILC2s **(F, G)** were quantified at 4dpe. Representative histogram depicting the percentage of cells vs. Bodipy signal is shown - Bodipy negative ILC2s (light grey), pPreF-Veh ILC2s (dark gray), and pPreF-RSV (red) **(G)**. Data are represented as mean ± SEM (n=2-7 mice per group). Statistical significance was calculated using an unpaired t-test **(B–F)**. Statistical significance was calculated using an unpaired t-test. **p ≤ 0.01. Created with BioRender.com.

**Figure 3:**
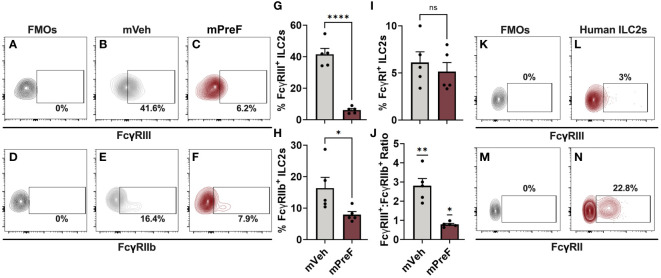
FcγRs are expressed on murine and human airway ILC2s. mVeh and mPreF offspring were generated and infected as described in **Figure 1**; all dams were immunized with PreF adjuvanted with alum. At 4dpe, right lungs were harvested for analysis of FcγRIII **(A–C, G)**, FcγRIIb **(D–F, H)**, and FcγRI **(I)** expression. Fluorescence minus one (FMO) controls were used to determine positive FcγRIII and FcγRIIb staining **(A,D)** and representative contour plots from mVeh and mPreF offspring are shown **(B, C, E, F)**. The frequencies of FcγRIII^+^ **(G)**, FcγRIIb^+^ **(H)** and FcγRI^+^ **(I)** ILC2s were quantified and the ratio of FcγRIII^+^:FcγRIIb^+^ was calculated **(J)**. The nasopharyngeal lavage fluid of pediatric patients undergoing adenoidectomies (n=3) was analyzed for ILC2 expression of FcγRIII **(K, L)** and FcγRII **(M, N)**. FMO controls were used to determine positive FcγRIII **(K)** and FcγRII staining **(N)**. Data are represented as mean ± SEM (n=5 mice per group). Statistical significance was calculated using an unpaired t-test **(G–I)** or Wilcoxon signed rank test **(J)**. ns, non-significant, *p ≤ 0.05, **p ≤ 0.01, and ****p ≤ 0.0001.

Animal studies were carried out in accordance with the University of Pittsburgh’s IACUC guidelines for the use and care of laboratory animals. Female Balb/cJ mice (7-8 weeks of age; The Jackson Laboratory, Bar Harbor, ME) were primed one week prior to breeding via intramuscular (IM) hind-limb injection with 50μL of PBS alone or the stabilized RSV prefusion F protein, DS-Cav1 (10μg per mouse; a gift from Jason McLellan and supplied by Calder Biosciences, Brooklyn, NY) formulated with Alum (100μg/mouse; Alhydrogel, *In vivo*gen; **Figures 1B–D**, **2**, **3**, **Supplementary Figures S1A, B**) or Advax (1mg/mouse; Vaxine, Pty, Ltd; **Figures 1E–H**, **Supplementary Figure S1C**). One week later, mice were bred, as previously described (4) and in the second week of gestation (3 weeks post-prime), mice were boosted IM with their respective vaccine. Offspring born to PBS-vaccinated dams are referred to as mVeh, while those born to adjuvanted DS-Cav1 vaccinated dams are labeled as mPreF.

For primary RSV infections, mice were intranasally (i.n.) infected with RSV L19 (provided by Dr. Martin Moore) at an infectious dose of 5x10^5^ plaque forming units (PFU) per gram of body weight at post-natal day (PND) 5-6 under isoflurane anesthesia, as previously described (4). At 1- and 4-days post-exposure, mice were culled using 100% isoflurane and cervical dislocation. RSV L19 was propagated and viral titers quantified as previously described (5).

For fatty acid uptake analysis, 50μg of Bodipy FL C_16_ (ThermoScientific) resuspended in DMSO was administered via intraperitoneally 60 minutes prior to sacrifice, as previously described (6).

### 2.2 RSV-neutralizing antibody – Renilla luciferase RSV reporter assay

Pre-challenge blood was collected via terminal bleed from non-exposed infants at PND5-6. In adult offspring, pre-challenge blood was collected via submandibular bleed immediately prior to RSV exposure and separated using Gel-Z serum separator tubes (Sarstedt). Serum was stored at -80°C until heat-inactivation (56°C for 30 minutes) and Renilla Luciferase RSV Reporter Assay was performed (7–10). Briefly, heat-inactivated serum was serially diluted in phenol-free MEM supplemented with 5% FBS and Pen/Strep before incubation in 96-well plate format with 100 PFU/well RSV L19-Renilla Luciferase virus (a gift from Martin Moore) for 2 hours at 37°C/5% CO_2_. After incubation, HEp-2 cells were trypsinized and a total of 2.5x10^4^ cells were added to 25μL FBS+Pen/Strep-containing phenol-free MEM and incubated for 64-66 hours at 37°C/5% CO_2_. After incubation, luciferase readouts were obtained using the Renilla-Glo Luciferase Assay system (Promega), according to manufacturer’s instructions. After incubation, luciferase activity was measured using a Novostar plate reader. The RSV luciferase assay generates a sigmoidal luminescence readout from which the midpoint (IC50) is calculated by nonlinear regression. The limit of quantification for the neutralization assay is set at the midpoint between the highest dilution and the next dilution in the 3-fold dilution series, as described previously (7–10). Serum dilutions were used to generate a full sigmoidal-shaped luminescence curve for the vaccinated animals and control samples are diluted equivalently to allow for cross-comparison. The limit of blank (cells only control) was previously determined for this assay to be 1826 RLU and the average of the virus-only control lanes included on each plate in the assay was 60,630 RLU. Since no Veh control samples achieved a signal less than 80% of the virus-only control even at their highest concentration, no IC50 could be determined for these samples and the lowest serum dilution used in the assay is reported as the neutralization titer, in this case 1:100 (marked by dashed line). All plates were run in duplicate and averaged.

### 2.3 Cell preparation, stimulation, and flow cytometry

Right lung lobes were collected, processed, and enumerated, as previously described (11). For ILC2 stimulation, lung homogenate was stimulated with PMA (30 ng/mL, Sigma Aldrich), ionomycin (500 ng/mL, Sigma Aldrich), and Brefeldin A (1:1000) in 10% RPMI at 37°C for 3 hours. Following stimulation, lung cells were surface stained, then fixed and permeabilized using BD Cytofix/Cytoperm™ kit (BD Biosciences) prior to intracellular cytokine staining. ILC2s were identified as previously described (3). Specifically, lung cells were surface stained with CD16/32 (Fc block; 2.4G2), LIVE/DEAD™ Fixable Blue Dead Cell Stain Kit, Lineage Cocktail (CD3 (17A2), Ly6G/Ly6C (RB6-8C5), CD11b (M1/70), CD45R (RA3-6B2), TER-119 (Ter-119)), CD49b (DX5), CD45 (30-F11), ST2 (DIH9), ICOS (C398.4A), CD127 (A7R34) and CD25 (PC61) and intracellularly stained for IL-5 (TRFK5) and IL-13 (ebio13A), as previously reported (3). Samples were run on a BD Fortessa or Cytek Aurora managed by the United Flow Core of the University of Pittsburgh. Data was analyzed using FlowJo V10 software (FLOWJO, LLC, OR).

### 2.4 ILC2 isolation and culturing

Right and left lungs from naïve adult mice were excised, minced, and digested in digestion media (Liberase, 2.5mg/500μL, MilliporeSigma; DNase, 1000μg/mL, Sigma Aldrich; Collagenase, 1mg/mL) for 45 minutes at 37°C/5% CO_2_. For complete homogenization, digested lungs were pushed through a 70μM mesh strainer and washed with HBSS. Lungs were then passed through a 40μM mesh strainer to generate a single cell suspension and subjected to ACK lysing for removal of red blood cells. Cells were then combined and resuspended in cILC2 media (RPMI 1640, 10% FBS, 100U/mL Pen/Strep, 1x non-essential amino acids (Gibco), 1mM sodium pyruvate (Lonza), supplemented with 10ng/μL recombinant IL-2 (rIL2, BioLegend). Cells were then seeded into 150mm petri dishes and rested for 3-4 hours at 37°C/5% CO_2_, allowing for removal of adherent epithelial and innate immune cells. After incubation, enriched non-adherent cells were collected and enumerated before surface staining with ILC2 sorting antibody cocktail, as previously described, in the presence of Fc block, as previously described (3). Cells were not fixed, as they were to be used for ex vivo stimulation studies. Cells were sorted on a FACS Aria with the assistance of the University of Pittsburgh United Flow Core personnel and defined as CD45^+^ Lineage^-^ CD49b^-^ CD117^-^ CD127^+^ ST2^+^, as previously described (2). Cells were sorted into cILC2 media supplemented with 50% FBS, to reduce cell shearing. After sorting, cells were enumerated and seeded into 96-well plates at a concentration of 5,000 ILC2s/well and rested overnight at 37°C/5% CO_2_ in cILC2 media until stimulation.

### 2.5 Immune complex generation and ex vivo ILC2 stimulation

To prepare Ab:RSV immune complexes (ICs), working stock RSV L19 was diluted in cILC2 media to achieve a concentration of 25,000 PFUs/60μL. Heat-inactivated serum from PreF/Alum-vaccinated dams was pooled and diluted in cILC2 media to a final dilution of 1:10. This optimized serum dilution was determined by incubating various dilutions of PreF/Alum serum with 25,000 PFUs of RSV and overlayed onto confluent HEp-2 cells and performing a standard plaque assay as previously described (5). A 1:10 dilution of pooled Alum serum resulted in undetectable RSV plaques and was the optimized serum dilution used for IC generation. To a 96-well flat-bottom plate (Corning), diluted PreF/Alum serum was incubated with 25,000 PFUs for 1 hour at 37°C/5% CO_2_ with intermittent shaking. At the end of incubation, pre-formed Ab : RSV ICs were added to rested ILC2s alone or in combination with rIL-33 (10ng/mL, BioLegend). Stimulated ILC2s were incubated at 37°C/5% CO_2_ for the indicated days; supernatants were then harvested and preserved for cytokine quantification.

To prepare Ova ICs, purified ovalbumin (Ova, Invitrogen) was incubated with purified mouse anti-Ova IgG1 (BioLegend), as previously described (12, 13). Briefly, Ova was incubated with mouse anti-Ova IgG1 at the indicated concentrations for 30 minutes at 37°C/5% CO_2_. Pre-formed Ova ICs were then added to sorted and rested ILC2s and incubated at 37°C/5% CO_2_ for 5 days, at which time supernatants were collected for cytokine quantification.

### 2.6 Cytokine quantification

Cytokine measurements from ILC2 supernatants were performed using the LEGENDplex™ Th Subpanel 3 Mix and Match Flow Immunoassay Kit (BioLegend), per manufacturer’s protocol. The top standard concentration for IL-5 and IL-13 quantification are 11ng/mL and 12ng/mL, respectively.

For IL-33 quantification from infant lungs, upper right lungs (URL) were excised, snap-frozen in liquid nitrogen, and stored at -80°C. For homogenization, URL was thawed on ice and homogenized in T-PER Tissue Protein Extraction Reagent (ThermoFisher) in the presence of a protease inhibitor cocktail (Promega). Homogenate was then centrifuged at 10,000rpm for removal of cellular debris. Cytokine quantification from lung homogenate was performed using the LEGENDplex™ Panel 2 Mix and Match Flow Immunoassay Kit (BioLegend), per manufacturer’s protocol. The top standard concentration used for IL-33 quantification was 50ng/mL.

Samples were acquired on a Cytek Aurora configured for bead detection, per manufacturer’s guidelines. Data was analyzed in the LEGENDplex Data Analysis Software Suite, per developer’s instructions. A 5-parameter logistic regression curve fitting algorithm was employed to generate a best fit standard curve for each analyte, which was then used to calculate analyte concentrations in experimental samples. R^2^ values for IL-33 = 0.9993; for IL-5 = 0.9978; and for IL-13 = 0.9895.

### 2.7 IL-33 western blot

Protein concentrations from URL homogenate were determined using the Bradford Assay (TCI America) according to manufacturer’s instructions. Samples were diluted 3:1 in 4X Laemmli buffer (Biorad) with 50nM DTT and boiled for 5 minutes. 30μg of protein was loaded per well on a 10% TGX Fastcast Acrylamide Gel (Biorad). Gels were run at 100V for 10 minutes before voltage was increased to 150V for an additional 45 minutes. Gels were then transferred to Immobilon-PSQ PVDF Membrane (MilliporeSigma) on ice for 1 hour. Membranes were blocked in 2.5% BSA-PBS for 1 hour at room temperature and then incubated with primary antibodies targeting β-actin (8H10D10, Cell Signaling Technology) and IL-33 (Nessy-1; Enzo Life Sciences) diluted in 2.5% BSA-PBS overnight at 4°C. Blots were then washed 4 times for 10 minutes in 0.1% Tween-PBS and incubated with HRP-conjugated secondary antibodies (rabbit anti-mouse IgG-HRP, Jackson Immunoresearch Laboratories; goat anti-rabbit IgG-HRP, Biorad) diluted in 2.5% non-fat milk-PBS for 1 hour at room temperature. Blots were washed as described previously and then incubated in Peirce ECL Western Blotting Substrate (Thermo Scientific) or Supersignal West Femto Maximum Sensitivity Substrate (Thermo Scientific). Blots were then imaged on a LI-COR Odyssey Imager and densitometric readings were measured using LI-COR Image Studio software (LI-COR Biosciences).

### 2.8 Statistical analysis

Statistical analyses were performed with GraphPad Prism 9 software (GraphPad Software, La Jolla, CA). Results are displayed as the mean ± SEM. For most analyses, data are compared using an unpaired t-test. Neutralizing antibody data was analyzed by nonlinear regression to obtain IC50 values, which were compared between groups using an unpaired t-test. For nonparametric ratio data, a Wilcoxon signed rank test was performed to compare the median of each group to a theoretical median of 1. For multiple comparisons, a one-way analysis of variance (ANOVA) with Tukey’s multiple comparisons test was performed to evaluate statistical significance between groups. p values ≤0.05 were considered significant.

## 3 Results

### 3.1 ILC2s are activated in pups born to immunized dams despite complete protection from replicating RSV

We postulated that neutralizing maternal antibody would mitigate IL-33 production by preventing viral-mediated damage to offspring airway epithelium. To test this, offspring born to dams immunized with alum or advax adjuvanted prefusion RSV protein (mPreF) or vehicle only (mVeh) were challenged with RSV at postnatal day 7, as depicted in **Figure 1A**. Since mothers, not offspring, are immunized, adjuvant-mediated CD4 Th1 or Th2 skewing is not expected in offspring. Serum collected prior to viral challenge confirmed that mPreF compared to mVeh offspring had significantly higher levels of RSV neutralizing maternal antibody (**Figure 1B**). At 1 day post RSV exposure (1 dpe), lungs were harvested. As expected, mPreF offspring had undetectable RSV lung titers (**Figure 1C**) as measured by hematoxylin & eosin plaque assay. Despite protection from replicating virus, levels of IL-33, an ILC2 activating cytokine, were similar between mVeh and mPreF offspring (**Figure 1D**), suggesting that prevention of RSV replication by maternal antibody failed to reduce IL-33 production.

We previously demonstrated that ILC2 activation peaks 4 days after secondary RSV exposure (3) and postulated that ILC2 activation would also occur at least 4 days after a primary RSV challenge. Thus, the study was repeated to determine if ILC2 activation was altered in mPreF vs mVeh pups at 4 dpe. Once again, RSV lung titers were undetectable in mPreF vs mVeh offspring (**Figure 1E**). Though a small increased trend was observed in ILC2 numbers from mPreF vs mVeh offspring, total numbers were similar between the groups (**Figure 1F**). The frequency of IL-13+ ILC2s were also similar between groups (**Figure 1G**). However, the proportion of IL-5+ ILC2s was markedly elevated in mPreF compared to mVeh offspring (**Figure 1H**), despite the absence of replicating virus.

### 3.2 ILC2s are activated following passive transfer of RSV-neutralizing antibody

Following RSV exposure, RSV-specific matAb binds to and neutralizes the virus resulting in the formation of immune complexes (ICs). To confirm that the ILC2 activation observed in mPreF offspring was driven by matAb:RSV ICs, ILC2 activation was measured in adult mice after passive transfer of RSV-neutralizing antibody followed by RSV challenge. Briefly heat-inactivated serum was pooled from PreF/Alum-vaccinated dams and 200µl was intraperitoneally injected into naïve adult mice per group 1 day prior to intranasal RSV challenge. One hour prior to sacrifice on 4 dpe, mice were intraperitoneally injected with fluorescently labeled palmitate (Bodipy C_16_), a fatty acid necessary for ILC2 production of IL-5 and IL-13 (6); lungs were processed for flow analysis of ILC2s (**Figure 2A**). Compared to unimmunized adult mice (RSV), passively immunized mice (pPreF-RSV) had a greater than 2-fold reduction in virus (**Figure 2B**). The total number of lung ILC2s trended higher in the presence of pPreF:RSV ICs (**Figure 2C**) with a parallel increasing trend in IL-13+ and IL-5+ ILC2s (**Figures 2D, E**). The increasing frequency of internalized Bodipy (**Figures 2F–G**), further suggests an increase in fatty acid uptake to support cytokine production. These results support the findings from **Figure 1** that ILC2s are activated despite complete protection against RSV replication and further suggest that ILC2 activation occurs in the presence of matAb:RSV ICs. Due to the low number of mice per group, further studies are needed to confirm these results following passive delivery of RSV neutralizing antibody.

### 3.3 FcγRs are expressed on murine and human airway ILC2s

IL-5+ ILC2s were increased in mPreF vs mVeh offspring despite similar levels of IL-33, suggesting an alternative mechanism of ILC2 activation. FcγR expression has been detected on other members of the ILC family of innate cells (NK cells and ILC3s) but has not been reported on lung ILC2s (14–16). Thus, we investigated the expression of Fc gamma receptors (FcγR) on pulmonary ILC2s as a possible mechanism by which matAb:RSV ICs mediate ILC2 activation (**Figure 3**). Fluorescence minus one (FMOs) controls were used to identify positive FcγR staining (**Figures 3A, D**). Expression of activating (FcγRIII; **Figures 3A–C**) and inhibitory (FcγRIIb; **Figures 3D–F**) FcγRs was detected on the surface of murine lung ILC2s. In mPreF offspring, the presence of matAb : RSV ICs was associated with a significant reduction in the frequency of ILC2s expressing FcγRIII and FcγRIIb compared to mVeh offspring (**Figures 3G–H**). Reduced expression suggests internalization of IC-bound FcγR following ligation by matAb : RSV ICs and is consistent with ligation-mediated FcγR internalization described in other innate cells (17–19). The strength of FcγR responses is largely determined by the ratio of activating to inhibitory receptors (20). Importantly, the ratio of activating FcγRIII to inhibitory FcγRIIb expression was markedly reduced in mPreF vs. mVeh offspring, suggesting greater internalization of activating FcγRIII (**Figure 3J**). FcγRI expression was not altered by matAb:RSV ICs (**Figure 3I**), aligning with reports that FcγRI does not bind IgG1, the predominant isotype generated following vaccination with Alum adjuvant (21). Because, FcγR expression differs between mice and humans (22), we asked if FcγRs are expressed on human ILC2s. ILC2s harvested from the lavage fluid of pediatric patients undergoing adenoidectomies expressed low levels of activating FcγRIII and high levels of inhibitory FcγRII (**Figures 3K–N**), suggesting a functional role for FcγR on human ILC2s. Together this data describes the first known report of activating and inhibitory FcγR expression on murine and human ILC2s and suggests a potential alternative mechanism of ILC2 activation following ligation and internalization of FcγRIII by matAb:RSV ICs.

### 3.4 Ex vivo stimulation with Ab:RSV ICs attenuates IL-33-mediated ILC2 activation

**Figure 4:**
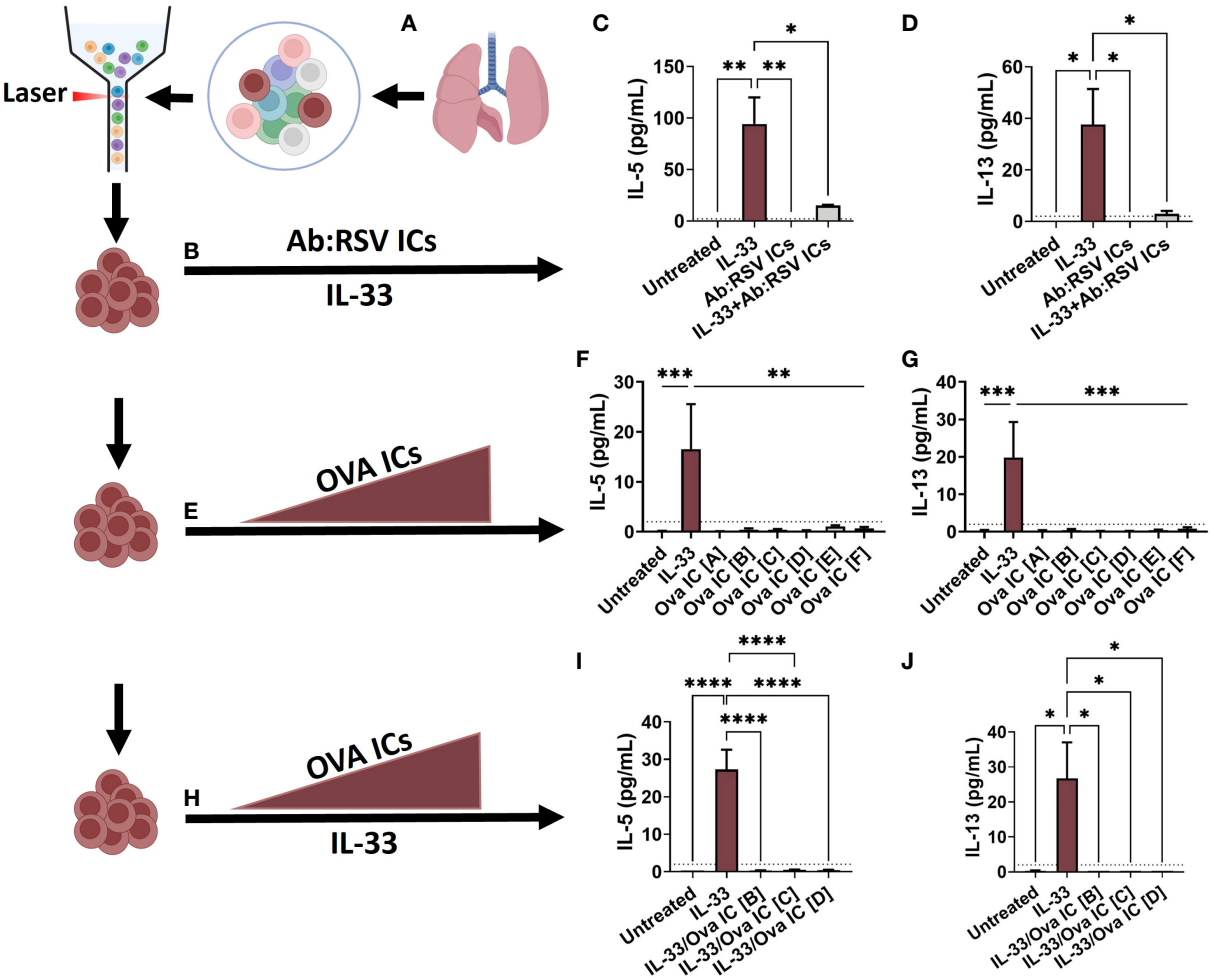
Ex vivo stimulation with Ab : RSV ICs attenuates IL-33-mediated ILC2 activation. ILC2s, defined as live, CD45^+^, Lin^-^, CD49b^-^ CD117^-^ CD127^+^ ST2^+^, were sorted from the lungs of naive adult mice and seeded into 96-well plates **(A)**. Cells were left untreated or stimulated with pre-formed Ab:RSV ICs +/- IL-33 (10ng/mL) **(B)**. At 5dps, IL-5 **(C)** and IL-13 **(D)** were quantified from ILC2 supernatants. In a repeat experiment, cells were left untreated or stimulated with 10ng/mL of IL-33 or increasing concentrations of pre-formed Ova ICs at the following ratios of Ova (mg/mL) to Anti-Ova IgG1 (mg/mL): Ova IC [A] 32.5/22.5, Ova IC [B] 65/45, Ova IC [C] 97.5/67.5, Ova IC [D] 130/90, Ova IC [E] 195/135, Ova IC [F] 260/180 **(E)**. At 5dps, IL-5 **(F)** and IL-13 **(G)** were quantified from ILC2 supernatants. In a third ex vivo experiment, cells were left untreated or stimulated with IL-33 alone or in combination with increasing concentrations of pre-formed Ova ICs at the following ratios of Ova (mg/mL) to A-Ova IgG1 (mg/mL): Ova IC [B] 65/45, Ova IC [C] 97.5/67.5, and Ova IC [D] 130/90 **(H)**. At 5dps, IL-5 **(I)** and IL-13 **(J)** were quantified from ILC2 supernatants. Dotted line represents the minimal detectable concentration. Data are represented as mean ± SEM (n=3-5 samples per group). Statistical significance was calculated using a one-way ANOVA with Tukey’s multiple comparison post-test. *p ≤ 0.05, **p ≤ 0.01, and ***p ≤ 0.01, ****p ≤ 0.0001. Created with BioRender.com.

To determine if ILC2s are capable of FcγR-mediated activation by Ab : RSV ICs, purified ILC2s were cultured with pre-formed Ab : RSV ICs and cytokine responses were measured. Briefly, naïve lungs were digested and processed for FACS sorting according to published protocols (23, 24). Purified ILC2s, defined as Live, CD45^+^, Lin^-^, CD49b^-^ CD117^-^ CD127^+^ ST2^+^, were then seeded into 96-well plates at a density of 5,000 cells/well and rested overnight (**Figure 4A**). One hour prior to stimulation, Ab : RSV ICs were generated by incubating heat-inactivated serum from DS-Cav1/PreF-vaccinated dams with RSV L19 at a serum dilution (1:10) and viral dose (2.5x10^4^ PFUs) optimized to neutralize RSV replication. ILC2s were left untreated or stimulated with IL-33 and/or Ab:RSV ICs and supernatants were collected for cytokine quantification at 5-days post-stimulation (dps; **Figure 4B**). As expected, IL-33 stimulation of ILC2s induced production of IL-5 and IL-13 (**Figures 4C, D**). Surprisingly, Ab:RSV ICs not only failed to induce IL-5 and IL-13 production, they significantly attenuated IL-33-induced IL-5 and IL-13 production (**Figures 4C, D**).

### 3.5 Increasing concentrations of Ova ICs fail to activate ILC2s

In mice, FcγRIIb has higher affinity for IgG1 than FcγRIII and at low concentrations, ICs comprised of IgG1 preferentially bind FcγRIIb and inhibit cell activation (25–27). As the concentration of ICs increase, so too does their engagement of the lower affinity activating FcγR, FcγRIII, ultimately shifting cellular responses (25). To test whether activation via FcγRIII was concentration dependent, cytokine production was quantified from purified ILC2s – as described in **Figure 4A** **–** and stimulated with increasing concentrations of Ova ICs (**Figure 4E**). Ova ICs were selected because, unlike RSV Ab ICs, the ratio of Ab to Ag could be controlled more precisely. Moreover, Ova ICs elicit cytokine production from FcγRII- and FcγRIII-expressing mast cells (28, 29), which, similar to ILC2s, orchestrate non-specific type 2 inflammation, including IL-13 production (13, 30). Ova ICs of various concentrations were generated by incubating known concentrations of anti-Ova IgG1 antibody and Ova antigen, such that the molar antibody:antigen ratio remained constant. The pre-formed Ova ICs were then added to rested ILC2s and supernatants were collected at 5dps for cytokine quantification. As expected, ILC2s exposed to IL-33 (positive control) elicited high levels of IL-5 and IL-13 (**Figures 4F–G**). However, high concentrations of Ova ICs failed to elicit detectable quantities of either cytokine (**Figures 4F–G**), suggesting that IgG1 ICs alone, even at higher concentrations, are not capable of activating ILC2s directly.

In the lungs of mPreF pups, IL-33 is present in similar quantities as those found in mVeh pups (**Figure 1D**). Thus, we postulated that Ova ICs may combine with IL-33 to enhance ILC2 cytokine production. To test this hypothesis, purified ILC2s were stimulated with IL-33 in the presence of 3 increasing Ova IC concentrations described in **Figure 4H**; cytokine levels were quantified at 5dps. Concentrations in the middle of the range [B-D], instead of the higher range [E-F] were chosen to improve our chances of seeing a dose-response when combined with IL-33. Similar to Ab:RSV ICs, high concentrations of Ova ICs reduced IL-5 and IL-13 production elicited by IL-33 alone (**Figures 4I–J**).

## 4 Discussion

The current understanding of ILC2 activation during RSV infection, is that ILC2s are activated by alarmins, including IL-33, released from airway epithelial cells in response to viral-mediated damage (31). Thus, high levels of RSV neutralizing matAb, would be expected to mitigate ILC2 activation (32). With similar IL-33 levels in mPreF vs mVeh pups, our novel discovery of FcγR expression on ILC2s poses a promising alternative mechanism of ILC2 activation in the absence of overt lung damage. It is important to note that the frequency of IL-5, but not IL-13, was increased in mPreF vs mVeh offspring (**Figures 1G, H**) and while both cytokines consistently trended up in adults treated with ICs, significance was not achieved due to low animal numbers (**Figures 2D, E**). Nonetheless, our ex vivo stimulation assay designed to interrogate FcγR function on ILC2s was unable to replicate the *in vivo* findings, and in fact, showed a suppression of IL-33-mediated ILC2 activation. A possible explanation for this discrepancy may be the amount of maternal antibody present *in vivo* vs ex vivo. While the titer of neutralizing maternal antibody present in infant mice at the time of RSV exposure is known, the quantity of matAb : RSV ICs is not known. Use of anti-Ova IgG1 was an approach to control the IC concentration used for ex vivo stimulation. However, the differences between the diverse population of antibodies generated during maternal immunization and that of the monoclonal anti-Ova antibody are extensive. For example, though both antibody pools are of the IgG1 subclass, the glycosylation patterns between the anti-DS-Cav1 antibody repertoire generated during maternal immunization and those of the monoclonal anti-Ova antibody are likely to differ tremendously (33). N-linked glycosylation is the most common post-transcriptional modification that functionally impacts IgG1 and can be altered by multiple factors, including sex, infection, and pregnancy (34). Additionally, the antibody:antigen ratio and size of ICs, which differs greatly between protein antigens (i.e. Ova vs DS-Cav1), impacts their mechanism of detection, extent of FcγR crosslinking, and downstream cellular responses (35–37). These factors complicate the translation of our *in vivo* findings to an ex vivo assay and may explain the failure of increasing Ova IC concentrations to stimulate ILC2s ex vivo. Future work will quantify matAb : RSV ICs present in the airways of mPreF pups to guide the generation of comparable quantities of ICs for ex vivo stimulation. Generation of ICs using anti-DS-Cav1 antibodies purified from the serum of directly vaccinated dams would also better mimic the antibody repertoire in mPreF pups and improve the interrogation of FcγR-mediated ILC2 activation. In addition to IL-33, other ILC2 activators (ex. TSLP, IL-25, PGD2) will be considered in future studies.

The complement system is a critical component of innate defense against pathogens and is rapidly initiated following detection of ICs (38). Specifically, when complement-fixing Abs, like IgG1 attach to viral surfaces, a cascade of events result in the conversion of C3 to C3a (39). In neonatal mice, the baseline concentration of C3 is markedly lower compared to adults and their ability to increase C3/C3a concentrations in response to IC detection is comparatively stunted (40). Interestingly, C3a is a mediator shown to directly activate ILC2s via ligation of C3aR (41). Because neonatal mice have reduced complement activity, our ex vivo stimulation assay used heat-inactivated serum to better mimic the neonatal environment demonstrated to foster ILC2 activation in the presence of matAb : RSV ICs (**Figure 4B**). However, the complete absence of complement may also explain the failure of ICs to stimulate ILC2s ex vivo. Future ex vivo stimulation assays will assess ILC2 activation in cultures containing low C3 concentrations and interrogate signaling events associated with C3aR ligation.

It is possible that cytokine production from ILC2s is not directly elicited by matAb:RSV ICs, but instead by cellular mediators elicited following IC-mediated activation of other immune cell populations. In support of this hypothesis is preliminary data in our model showing higher levels of cleaved IL-33 in mPreF vs. mVeh pups (**Supplementary Figures S1A, B**). Cleavage of full-length IL-33 into its more biologically potent form is mediated by inflammatory proteases produced by neutrophils and mast cells (42–44). The increase in cleaved IL-33 in mPreF pups suggests that matAb:RSV ICs drive early cellular responses not captured in our current studies that are responsible for neutrophil or mast cell activation and the subsequent proteolytic cleavage of IL-33. In fact, presence of ICs in the airways can lead to acute lung injury and subsequent complement-dependent neutrophil recruitment to the airways (45). Therefore, it is intriguing to hypothesize that matAb:RSV IC-mediated acute lung damage leads to the rapid influx of neutrophils providing the proteases necessary to generate cleaved IL-33 and activate ILC2s.

FcγR ligation on ILC2s may have alternative functions, such as mediating internalization of exogenous antigens (46). In addition to their cytokine producing effector cells, ILC2s are also capable of antigen presentation and CD4^+^ T cell activation (47–49). On traditional antigen presenting cells, ICs bind FcγRs, triggering their internalization and delivery to lysosomes, where they are processed and loaded into MHCII molecules for cell-surface presentation and CD4^+^ T cell priming/activation (50). In infants born to maternally vaccinated dams, the frequency of MHCII^+^ ILC2s is significantly higher in mPreF vs mVeh pups, providing compelling preliminary evidence for the antigen presentation function of FcγRs on ILC2s (**Supplementary Figure S1C**).

Collectively, this brief report describes changes in ILC2 activation and FcγR expression in the presence of neutralizing matAb that occur despite complete protection against RSV replication. To our knowledge, this is the first report of FcγR expression on lung ILC2s with differential expression in the presence of matAb. These data warrant further studies to better understand how ILC2s are differentially regulated in the presence of RSV-neutralizing maternal antibody despite protection against replicating virus. Understanding how matAb alters the infant immune response to virus is particularly important to minimize unwanted type 2 inflammation in early-life and ensure the long-term safety of maternal RSV vaccines.

## Data availability statement

The raw data supporting the conclusions of this article will be made available by the authors, without undue reservation.

## Ethics statement

The studies involving humans were approved by University of Pittsburgh Institutional Review Board. The studies were conducted in accordance with the local legislation and institutional requirements. Written informed consent for participation in this study was provided by the participants’ legal guardians/next of kin. The animal study was approved by University of Pittsburgh Institutional Animal Care and Use Committee. The study was conducted in accordance with the local legislation and institutional requirements.

## Author contributions

JK: Conceptualization, Data curation, Formal analysis, Funding acquisition, Investigation, Methodology, Project administration, Resources, Software, Supervision, Validation, Visualization, Writing – original draft, Writing – review & editing. KEi: Data curation, Investigation, Writing – review & editing. ML: Investigation, Methodology, Writing – review & editing. SG: Methodology, Writing – review & editing. DB: Methodology, Writing – review & editing. MY: Methodology, Writing – review & editing. DC: Resources, Writing – review & editing. TP: Formal analysis, Writing – review & editing. KEm: Conceptualization, Funding acquisition, Project administration, Resources, Supervision, Visualization, Writing – review & editing.
