# Supplementary material for: Lung ILC2s are activated in BALB/c mice born to immunized mothers despite complete protection against respiratory syncytial virus
Source: Front Immunol. 2024 May 17;15:1374818. doi: 10.3389/fimmu.2024.1374818 (PMC11140082; doi:10.3389/fimmu.2024.1374818)
Supplement: Supplementary file 1 [file Presentation_1.pptx]

## Slide 1
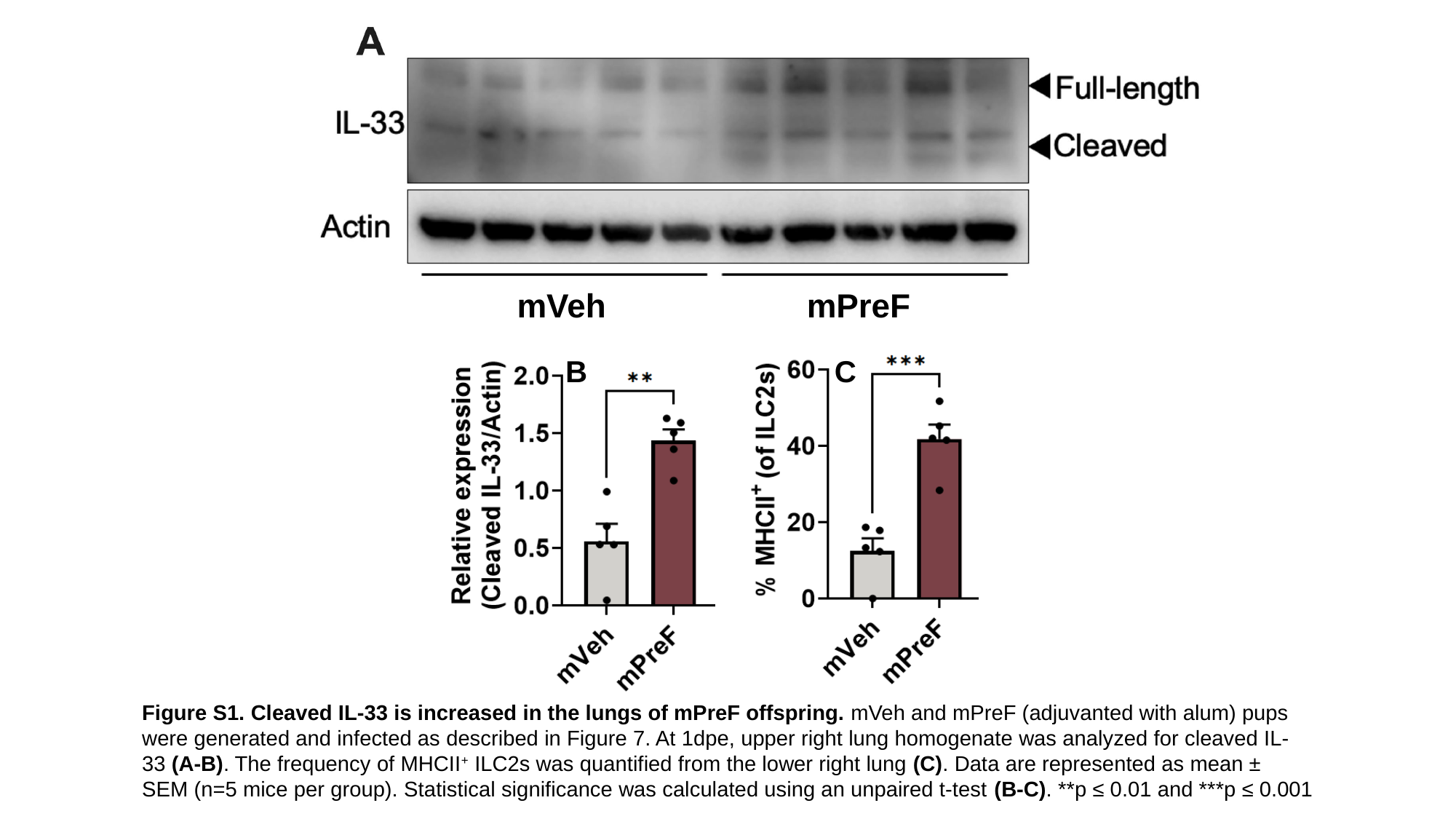

mVeh
mPreF
B
C
Figure S1. Cleaved IL-33 is increased in the lungs of mPreF offspring. mVeh and mPreF (adjuvanted with alum) pups were generated and infected as described in Figure 7. At 1dpe, upper right lung homogenate was analyzed for cleaved IL-33 (A-B). The frequency of MHCII+ ILC2s was quantified from the lower right lung (C). Data are represented as mean ± SEM (n=5 mice per group). Statistical significance was calculated using an unpaired t-test (B-C). **p ≤ 0.01 and ***p ≤ 0.001
